# Overactivation of the Endocannabinoid System in Adolescence Disrupts Adult Adipose Organ Function in Mice

**DOI:** 10.3390/cells13050461

**Published:** 2024-03-06

**Authors:** Kwang-Mook Jung, Lin Lin, Daniele Piomelli

**Affiliations:** 1 Department of Anatomy and Neurobiology, University of California, Irvine, CA 92697, USA; kmjung@uci.edu (K.-M.J.); linll1@hs.uci.edu (L.L.); 2 Department of Biological Chemistry, University of California, Irvine, CA 92697, USA; 3 Department of Pharmaceutical Sciences, University of California, Irvine, CA 92697, USA

**Keywords:** cannabis, adolescence, trans-differentiation, adipocyte

## Abstract

Cannabis use stimulates calorie intake, but epidemiological studies show that people who regularly use it are leaner than those who don’t. Two explanations have been proposed for this paradoxical finding. One posits that Δ^9^-tetrahydrocannabinol (THC) in cannabis desensitizes adipose CB1 cannabinoid receptors, stopping their stimulating effects on lipogenesis and adipogenesis. Another explanation is that THC exposure in adolescence, when habitual cannabis use typically starts, produces lasting changes in the developing adipose organ, which impacts adult systemic energy use. Here, we consider these possibilities in the light of a study which showed that daily THC administration in adolescent mice produces an adult metabolic phenotype characterized by reduced fat mass, partial resistance to obesity and dyslipidemia, and impaired thermogenesis and lipolysis. The phenotype, whose development requires activation of CB1 receptors in differentiated adipocytes, is associated with overexpression of myocyte proteins in the adipose organ with unchanged CB1 expression. We propose that adolescent exposure to THC causes lasting adipocyte dysfunction and the consequent emergence of a metabolic state that only superficially resembles healthy leanness. A corollary of this hypothesis, which should be addressed in future studies, is that CB1 receptors and their endocannabinoid ligands may contribute to the maintenance of adipocyte differentiation during adolescence.

## 1. Introduction

One of cannabis’ most peculiar and iconic effects is the stimulation of hedonic eating—the seeking of palatable foods in the absence of physical hunger [1]. This response, which is known in American popular culture as ‘the munchies’, results from an undue recruitment of the endocannabinoid system (ECS) by cannabis’ intoxicating constituent, Δ^9^-tetrahydrocannabinol (THC) [2,3]. The ECS is a homeostatic signaling complex composed of CB1 and CB2 cannabinoid receptors, endogenous lipid-derived ligands for those receptors (the endocannabinoids), and proteins involved in the formation, transport, and elimination of such ligands [4]. When cannabis is consumed, THC overrides the system’s normal function, producing a unique spectrum of physiological and psychological effects that range from changes in body temperature and blood pressure to alterations in mood and memory [5]. The fact that hedonic hunger is so prominent among such effects reflects the critical roles played by the ECS in the seeking and sensing of high-calorie foods [6,7]. On the other hand, the ability of CB1 receptor antagonists and inverse agonists—which act counter to THC and suppress intrinsic ECS activity—to reduce body weight and waist circumference and improve metabolic profile highlights the breadth of roles played by the ECS in the control of energy balance [8]. 

THC administration increases calorie intake in laboratory animals and humans [8,9] but, surprisingly, healthy people who consume cannabis on a daily or quasi-daily basis are leaner than non-users. Indeed, epidemiological surveys have almost unanimously shown that cannabis users have lower body mass index (BMI) compared to non-users and that habitual cannabis consumption is associated with lower BMI and improved cardiometabolic risk [10,11,12,13,14,15,16,17,18,19,20,21,22,23,24,25,26], which cannot be attributed to factors such as tolerance [27], lifestyle, or a propensity of lean persons to become cannabis users [16]. In this minireview, we summarize the evidence for this apparent paradox and discuss a possible cellular mechanism underpinning it: we propose that excessive CB1 receptor activation during adolescence, but not later in life, produces a dysfunction in the adult adipose organ which alters systemic energy utilization and results in a ‘pseudo-lean’ phenotype that only superficially resembles healthy leanness. From these findings follows the suggestion, which future studies should evaluate, that endocannabinoid signaling helps maintain adipocytes in a fully differentiated state as the body transitions through adolescence.

## 2. Habitual Cannabis Users Are Surprisingly Lean

The hunger-stimulating effects of THC are mediated by CB1 receptors located in multiple body structures, including hypothalamic neurons that control feeding [28,29,30,31,32] and small intestinal enterocyte-vagal circuits that regulate dietary fat intake [6,33]. It is likely that these same receptors are activated when people use cannabis and, conversely, are blocked during treatment with a CB1 receptor antagonist or inverse agonist. Yet, epidemiological studies unequivocally show that persons who consume cannabis are leaner than those who don’t (Table 1). Cross-sectional and longitudinal surveys indicate that healthy cannabis users have lower BMI compared to healthy non-users [10,11,12,13,14,15,16,17,18,19,20,21,22,23,24,25,26] and that cannabis consumption is inversely associated with BMI, waist circumference, and other cardiometabolic risk factors [10,11,12,13,14,15,16,17,18,19,20,21,22,23,24,25,26]. In 2001, the analysis of a dataset obtained from a cross-sectional survey of 10,623 participants, aged 20 to 59 years, from the National Health and Nutrition Examination Survey (NHANES III, 1988–1994) showed that BMI is significantly (*p* < 0.0001) lower in persons who reported using cannabis in the past month (8.7% of the sample) compared to those who did not report any use [22]. The difference in BMI remained significant (*p* < 0.003) even after adjusting for age, biological sex, education, cigarette smoking and, importantly, nutrient consumption [22]. In fact, calorie intake was higher in survey participants who consumed cannabis than in those who did not [16,22]. Furthermore, persons who regularly use cannabis were found to have smaller waist circumference and improved high-density lipoproteins, low-density lipoproteins, triglycerides, fasting glucose, insulin resistance, and blood pressure [11,12,23,24], as well as reduced risk of developing type 2 diabetes [20,21]. The longitudinal prospective study NESARC (National Epidemiologic Survey on Alcohol and Related Conditions), whose sampling was designed to match the adult US population, confirmed that cannabis use and BMI are negatively associated (Table 1) [17].

## 3. The ECS in the Adipose Organ

**Figure 1:**
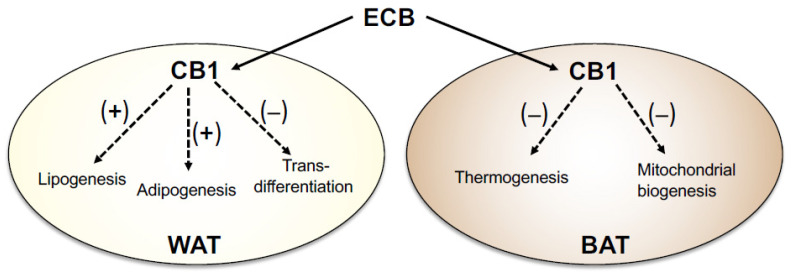
Endocannabinoid signals in the physiology of WAT and BAT. (**Left**) In WAT, CB1 receptor activation produced by endocannabinoid (ECB) ligands increases glucose uptake and fatty acid biosynthesis, catalyzed by fatty acid synthase (FAS), resulting in heightened lipogenesis. Increased ECB tone also enhances the transcription of genes involved in adipogenesis, such as the ligand-operated transcription factor, PPARγ, while suppressing mitochondrial biogenesis and trans-differentiation into beige/brown adipocytes. (**Right**) In BAT, ECB signaling reduces non-shivering thermogenesis triggered by β3-adrenergic receptor (ADRB3) activation. Blockade of CB1 receptors enhances thermogenesis by dampening local ECB activity and consequently boosting sympathetic outflow to the adipose organ. (+), increase; and (−), decrease.

To understand the paradoxical response to long-term cannabis use, we must first take a deeper dive into ECS biology. As pointed out above, this signaling complex is comprised of CB1 and CB2 receptors, their endogenous agonists anandamide and 2-arachidonoyl-sn-glycerol (2-AG), and various enzymes and transporter proteins responsible for the formation, transfer, and deactivation of such agonists [4,34]. All these elements are represented in white and brown adipose tissues (WAT and BAT, respectively)—the parenchymal components of the adipose organ [35]—where anandamide and 2-AG are thought to act as autocrine/paracrine messengers [6,36] to heighten lipogenesis and adipogenesis and attenuate mitochondrial biogenesis and non-shivering thermogenesis [37,38] (Figure 1). The critical roles played by the ECS in adipose homeostasis are underscored by the remarkable anti-obesity effects of agents that block intrinsic ECS activity—including globally active or peripherally restricted CB1 antagonists and inverse agonists [39]—which have been documented by numerous preclinical and clinical studies [40,41,42,43,44,45,46,47]. These agents can temporarily reduce nutrient intake in rodents and humans, but their anorexic effects disappear with time. Thus, more than in appetite suppression, the anti-obesity properties of CB1 antagonists and inverse agonists should be sought in their ability to stimulate lipolysis, energy dissipation, and fatty acid oxidation by halting endocannabinoid signals that promote energy storage in the adipose and other organs [39,47].

**Figure 2:**
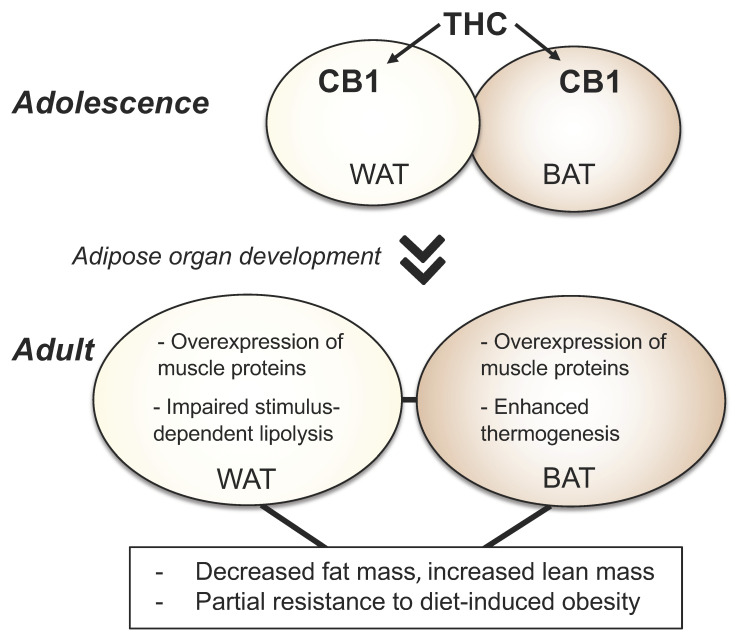
Effects of adolescent THC exposure on mouse adipocytes. Daily exposure to THC during adolescence causes an overactivation of CB1 receptors in differentiated AdipoQ-expressing adipocytes, resulting in ectopic overexpression of contractile proteins normally present in myocytes and dysregulation of two key adipocyte functions: lipolysis in WAT and thermogenesis in BAT. These molecular modifications may account, at least partially, for the adult phenotype of THC-exposed mice, whose features include decreased fat mass, increased lean mass, partial resistance to diet-induced obesity and dyslipidemia, abnormal thermogenesis, and blunted stimulus-induced lipolysis.

Like all plant-derived cannabinoids, THC is highly hydrophobic and accumulates in fat depots at concentrations that are much greater than those needed to fully engage local CB1 receptors [48,49]. When chronically activated, these receptors should promote, as described above, a metabolic phenotype characterized by higher-than-normal nutrient intake and adiposity, hyperlipidemia, and increased risk of diabetes. This assumption is supported by the orexigenic effects of the peripherally restricted CB1 receptor agonist ART27.13 [50]. The fact that habitual cannabis users display an opposite physical composition is a striking inconsistence that demands a mechanistic explanation. Two have been proposed. The first posits that prolonged THC exposure stops energy-saving ECS signals by promoting the desensitization and downregulation of adipose CB1 receptors [16]. There are, however, two difficulties with this account: first, people who regularly use cannabis do not seem to develop tolerance to the drug’s orexigenic effects [15,22,51] and, second, in mice sub-chronically treated with THC, adipose CB1 expression is not significantly affected [52]. Another possible, non-exclusive explanation is that excessive CB1 activation during the teenage years—when regular use of cannabis typically begins [11,53]—produces enduring physiological changes in the still-developing adipose organ, which impact systemic energy utilization in adulthood (Figure 2). In the following sections, we discuss the results of a recent study that support this possibility [52].

## 4. Adolescent THC Exposure Impacts Adult Systemic Metabolism

Daily intraperitoneal injections of THC in 30- to 43-day-old mice—a time that roughly corresponds to human adolescence [54]—cause a marked dampening of the animals’ weight-gain trajectory [52]. This effect was obtained with a THC dose (5 mg/kg) that exerts only modest physiological and behavioral effects in adolescent mice, most likely because at this developmental stage the mouse brain is partially impermeant to THC, as shown by the finding that intraperitoneal injections of THC (5 mg/kg) produced brain concentrations and a brain-to-plasma ratio of the drug that were 40–60% lower in adolescent relative to adult animals [49,55]. This may be at least partly due to the fact that the brains of adolescent mice contain higher mRNA levels of the multidrug transporter breast cancer resistance protein, which may extrude THC from the brain, and higher mRNA levels of claudin-5, a protein that contributes to blood-brain barrier integrity [49]. 

Immediately after the end of adolescent THC treatment, when the drug was cleared from brain tissue but was still present in fat depots, the mice expended more energy than did vehicle-treated controls. Even more notably, as they reached young adulthood (PND70) and completely eliminated the drug, THC-treated animals transitioned to an abnormal metabolic state whose features included decreased fat mass and white adipocyte area, increased lean mass, partial resistance to diet-induced obesity and dyslipidemia, enhanced thermogenesis, and blunted lipolysis. The latter two effects are especially noteworthy because they are suggestive of a pervasive impact on the body’s capacity to manage energy and nutrients. In THC-exposed mice, baseline body temperature was higher and the thermogenic response to cold or pharmacological adrenergic stimulation was all but suppressed. Lipolysis was similarly impaired, which raises the intriguing possibility that adolescent THC exposure might deprive the adult brain of critical adipose-derived nutrients which are mobilized by sympathetic activation under conditions of social or environmental stress. 

The emergence of this distinctive phenotype depends on CB1 receptor activation in differentiated adipocytes of adolescent mice. Three sets of experiments support this conclusion [52]. First, when young-adult animals (PND 70-83) received the same THC treatment given to adolescents, their body-weight trajectory remained unchanged, and the limited transcriptional modifications observed in their adipose organ did not include expression of muscle-related genes. Second, the change in body-weight gain induced by THC was prevented by co-administration of a global CB1 receptor inverse agonist (AM251) or a peripheral CB1 neutral antagonist (AM6545), but not by the CB2 inverse agonist AM630. Lastly, and importantly, no such change occurred in adolescent mice lacking CB1 receptors in adipocytes that express the protein hormone adiponectin, which are thought to be fully differentiated [56,57]. 

Collectively, the findings outlined above suggest that daily exposure to THC in adolescence promotes—through a developmentally regulated mechanism that requires CB1 activation in differentiated adipocytes—a lasting state that resembles healthy leanness but results in fact from a dysregulation of stimulus-induced lipolysis and thermogenesis, two basic functions of the mature adipose organ. We refer to such a state as ‘pseudo-lean’.

### 4.1. Alterations in Gene and Protein Expression

At the molecular level, one notable feature of this pseudo-lean phenotype is the lack of any substantial abnormality in the ECS components of BAT or WAT [52]. For example, CB1 receptor mRNA and protein or mRNAs encoding for the 2-AG-metabolizing enzymes, diacylglycerol lipase-α (DGL-α) and monoglyceride lipase (MGL), were not affected in the BAT or WAT of THC-exposed mice. This argues against the suggestion that ECS disfunction—e.g., blunted CB1 signaling or endocannabinoid activity—might account for the state’s long-term maintenance [16]. To search for an alternative explanation, we carried out unbiased transcriptomic analyses of BAT and WAT from control and THC-treated mice. These revealed that a surprisingly large fraction of the adult adipose transcriptome—2985 genes in BAT and 3446 in WAT—was altered by adolescent exposure to the drug. One particularly salient alteration was the ectopic expression of transcripts encoding for contractile proteins that are normally found in smooth and striated muscle. These included sarcomere constituents—e.g., titin (Ttn), myosin heavy chain (Myh), myosin light chain (Myl), troponin 1 (Tnni2), and troponin T (Tnnt3)—as well as muscle-associated enzymes—e.g., enolase 3 (Eno3) and sarco(endo)plasmic reticulum calcium ATPase 1 (Atp2a1). Conversely, genes involved in mitochondrial respiration and protein synthesis were significantly downregulated, including those encoding for proteins of the mitochondrial matrix and inner membrane. These molecular modifications were both developmentally controlled and organ-specific, as they were not seen after exposure to THC in young adulthood and were not matched by similar changes in skeletal muscle, where the transcription of genes implicated in muscle structure and function was instead suppressed. 

Untargeted proteomic experiments confirmed and extended the transcriptomic findings [52]. Both BAT and WAT were found to contain large quantities of sarcomere proteins, which in BAT included titin (30-fold increase over control), troponin (2-fold), and myosin (1.5-fold). Importantly, immunohistochemical staining of intrascapular BAT demonstrated that, in THC-treated mice, titin was highly expressed in adipocytes whereas, in control animals, it was only found in vascular smooth muscle, as expected for a sarcomere-associated protein. Electron microscopy studies showed, however, that the ectopic expression of muscle proteins did not detectably alter adipocyte ultrastructure; for example, lipid droplet size remained unchanged.

### 4.2. Alterations in Metabolism

Adolescent THC treatment did not significantly impact the WAT metabolome [52], but the concentrations of several metabolic products—including essential, non-essential, and N-acetylated amino acids—were increased in BAT. The NADPH/NADP+ ratio was significantly decreased, whereas no change was detectable in metabolites related to energy production. One possible explanation for this profile, which is consistent with the transcriptomic and proteomic data outlined above, is that adipocytes in the adult BAT of THC-exposed mice overconsume NADPH (thus lowering the NADPH/NADP+ ratio) for the biosynthesis of ectopically expressed myocyte proteins.

## 5. Conclusions

Cannabis’ intoxicating constituent, THC, stimulates nutrient intake and energy conservation by engaging CB1 receptors in the brain, adipose, and other organs. Nevertheless, epidemiological studies have consistently shown that people who frequently use cannabis are leaner than non-users. Here, we outline a possible molecular explanation for this paradoxical finding. Based on the results of omics studies conducted in mice, we propose that overactivation of adipocyte CB1 receptors during adolescence—when the adipose organ is still developing, and habitual cannabis use often starts—reprograms transcription in adult BAT and WAT adipocytes to induce the ectopic expression of myocyte-associated proteins such as titin and troponin. The cellular resources needed for the execution of this non-physiological task are subtracted from those available for normal mRNA and protein synthesis, resulting in the lasting disruption of two critical adipocyte functions—stimulus-induced lipolysis and thermogenesis—and in the emergence of a lasting pseudo-lean phenotype that is strikingly reminiscent of the one exhibited by habitual cannabis users. This hypothesis does not rule out the possible contributions made by other organ systems that participate in energy metabolism, such as skeletal muscle and the brain, which may be affected by adolescent THC exposure. Indeed, our own work has found significant transcriptional changes, in both the muscle and brain of mice exposed to THC in adolescence [52,58], which deserve further investigation. 

## 6. Future Directions and Perspectives

The broad impact of early-life exposure to cannabis remains understudied. Understanding the molecular underpinnings of enduring alterations brought about by the drug and their possible link to adolescent exposure is necessary to fill this knowledge gap, inform evidence-based prevention, and guide future investigations. Two, among several, stand out. First, the finding that the lasting impact of CB1 overactivation is developmentally controlled suggests that a delicate balance of ECS signals, disrupted by frequent THC exposure, may preserve adipocytes in a fully differentiated state within the volatile physiological context of adolescence. Future pharmacological, neurohormonal, and epigenetic studies should explore this possibility. Second, the striking increase in titin expression caused by adolescent THC treatment raises the intriguing possibility that this protein may be more than a passive contributor to adipose dysfunction. Indeed, while titin’s main role is to provide elasticity and stability to sarcomeres, studies have shown that this giant protein also contains a kinase domain [59] and may serve as a hub for signal transduction [60]. It is thus reasonable to speculate that changes in titin expression, such as those observed in our study, might have pervasive consequences for transcription regulation or related processes. 

## Figures and Tables

**Table 1:** Clinical studies on obesity, BMI, and other metabolic risk factors in healthy cannabis users.

Study Design (Publish Year)	Number of Subjects (% Male) /Age (years)	Participants/Data Collection	Main Effects of Cannabis on BMI and Obesity (Significance)	Other Metabolic Changes Observed in Cannabis Users	Ref.
Ongoing prospective study (2022)	3706 (63% male) /31 to 58	French ANRS CO22 Hepather cohort	Current cannabis use is associated with lower risk of central obesity assessed by waist circumference (*p* = 0.001).	Lowered risk of overweight and obesity assessed by high BMI.	[14]
Prospective, up to 2-year follow-up (2020)	401 (54.1% male) /14 to 17	Recruited from Miami-Dade County middle and high schools	Negative association between cannabis use and BMI slope (*p* = 0.004).	Baseline BMI predicted a positive and significant association with cannabis use slope.	[26]
Prospective, follow-up over 25 years (2019)	253 (100% male) /7 to 32	The youngest cohort of the Pittsburgh Youth Study (PYS)	Lower BMI (*p* < 0.001) and other cardiometabolic risk factors in cannabis users.	Smaller waist-hip ratio, better HDL, LDL, TG, fasting glucose, insulin resistance, and BP in cannabis users.	[11]
Prospective, 3-year follow-up (2019)	33,000 (45% male) /18 years and older	Residents in all US states, National Epidemiologic Survey on Alcohol and Related Conditions (NESARC)	Inverse association between cannabis and BMI increase (*p* < 0.001, adjusted for age).	77% of the participants never used cannabis, 18% had discontinued use (‘quit’), 3% were initiates, and 2% were persistent users.	[17]
Prospective, 8-year follow-up (2016)	17,833 (42% male) /18 to 84	The Stockholm Public Health Cohort (SPHC)	Recent cannabis users had lower levels of BMI at baseline (ORs 0.68, 95% CIs 0.47–0.99).	The crude association showed that cannabis users had a reduced risk of type 2 diabetes, but it was not significant after adjusting for age.	[20]
Prospective, follow-up over 20 years (2016)	1037 (51.6% male) /18 to 38	The Dunedin Multidisciplinary Health and Development Study (New Zealand)	No clear association observed.	Poorer periodontal health.	[10]
Analysis on dataset from national survey (2016)	Two cohorts of 13,038 and 13,972 /adolescence	National Longitudinal Survey of Adolescent Health, Waves III and IV	Reductions in BMI (*p* < 0.01 for both female and male) in cannabis users.	Alternative measures of body size (waist circumference) decreased in cannabis users.	[12]
Analysis on a cross-sectional survey (2015)	6281 (43% male) /20 to 59	National Health and Nutrition Examination Survey (NHANES, 2005–2012)	Statistically significant associations between past and current use of cannabis with lower level of BMI (*p* < 0.0001).	Lower fasting insulin, insulin resistance, and waist circumference in cannabis users compared with nonusers.	[24]
Cross-sectional, case-control study (2013)	30 (60% male) /27 ± 8	NIH/the Johns Hopkins Behavioral Pharmacology Research Unit (BPRU) (NCT00428987)	Higher percent abdominal visceral fat (*p* = 0.004), lower plasma HDL cholesterol (*p* = 0.02), and lower adipocyte insulin sensitivity index (*p* < 0.05) in cannabis users.	Not observed are hepatic steatosis, insulin insensitivity, impaired pancreatic beta-cell function, or glucose intolerance.	[15]
Analysis on a cross-sectional data (2013)	815 (40% male) /Mean age of 32	African American and Puerto Rican young adults who in 1990 attended schools serving East Harlem, NYC	More frequent cannabis use was associated with a lower likelihood of obesity (*p* < 0.05).	Protective factors such as physical activity, healthy diet, self-control, and life satisfaction were associated with a reduced probability of being obese.	[19]
Analysis on a cross-sectional survey (2013)	4657 (49% male) /20 to 59	Data from National Health and Nutrition Examination Survey (NHANES, 2005–2010)	Statistically significant associations between past and current use of cannabis with lower level of BMI (*p* < 0.0001).	Current cannabis use is associated with lower levels of fasting insulin, lower homeostasis model assessment of insulin resistance (HOMA-IR), and smaller waist circumference.	[23]
Analysis on a cross-sectional survey (2012)	10,896 (42.9% male) /20 to 59	Data from the National Health and Nutrition Examination Survey (NHANES III, 1988–1994)	Cannabis users had lower rates of obesity (BMI over 30) and lower mean BMI (*p* < 0.001), with current heavy marijuana users having the lowest BMI.	Cannabis users had a lower age-adjusted prevalence of type 2 diabetes mellitus (DM) compared to non-users (*p* < 0.0001).	[21]
Analysis on two cross-sectional data from 2 national survey (2011)	NESARC, 41,633 (43.6% male); and NCS-R, 9103 /18 years or older	The National Epidemiologic Survey on Alcohol and Related Conditions (NESARC) and the National Comorbidity Survey– Replication (NCS-R)	The prevalence of obesity was significantly lower in cannabis users than in nonusers in both studies (*p* < 0.001 for both).	The lowered prevalence of obesity in cannabis users was not accounted for by tobacco smoking status.	[18]
Prospective, 21-year follow-up of mothers and the children (2010)	2566 (49% male) /18.2 to 23.1	Mater-University of Queensland Study of Pregnancy (MUSP)	Positive association between frequent cannabis use and less overweight/obesity (*p* < 0.001) in everyday cannabis user cohort).	Those who had used cannabis were less likely to be categorized in the BMI ≥ 25 group.	[13]
A cross-sectional study (2005)	297 (0% male) /16 to 79	Patients for morbid obesity/weight management	Negative correlation between BMI group and percent cannabis use (*p* = 0.0173).		[25]
Analysis on a cross-sectional survey (2001)	10,623 (47% male) /20 to 59	Data from the National Health and Nutrition Examination Survey (NHANES III, 1988–1994)	BMI was lower in the heavy cannabis users than in the non-current users (*p* < 0.0001) and these differences remained after adjusting for age, gender, education, cigarette smoking, and caloric intake (*p* < 0.003).	Current cannabis users had higher intakes of energy and nutrients than non-current cannabis users.	[22]
Meta-analysis of 17 studies (2018)	78,331 (various)	Various	Reduced BMI (*p* < 0.0005) and rates of obesity (*p* < 0.05).	Caloric intake is increased in cannabis users.	[16]

## Data Availability

Not applicable.
